# Percutaneous sacroiliac screw fixation with a 3D robot-assisted image-guided navigation system: Technical solutions

**DOI:** 10.1007/s00064-024-00871-9

**Published:** 2024-11-18

**Authors:** Björn-Christian Link, R. A. Haveman, B. J. M. Van de Wall, R. Baumgärtner, R. Babst, F. J. P. Beeres, P. C. Haefeli

**Affiliations:** Klinik für Orthopädie und Unfallchirurgie, Luzerner Kantonsspital, Spitalstrasse, 6000 Luzern, Switzerland

**Keywords:** Fragility fractures, Three-dimensional robot-assisted image-guided navigation system, Sacroiliac screw osteosynthesis, Hybrid surgical operating room, Minimally invasive surgical procedures, Fragilitätsfrakturen, Robotergestütztes bildgesteuertes 3D-Navigationssystem, Sakroiliakale Schraubenosteosynthese, Hybridoperationssaal, Minimal-invasive Operationsverfahren

## Abstract

**Objective:**

Presentation and description of percutaneous sacroiliac (SI) screw fixation with the use of a 3D robot-assisted image-guided navigation system and the clinical outcome of this technique.

**Indications:**

Pelvic fractures involving the posterior pelvis.

**Contraindications:**

Patients not suited for surgery.

**Surgical technique:**

Planning the screws on the diagnostic computer tomogram (CT). Matching with a low-dose CT in the operating room. Lateral incision. Verify the guidewire position with the personalized inlet and outlet views. After correct positioning, place a cannulated screw over the guidewire. For fragility fractures, augmentation is recommended. Finish the surgery with a final 3D scan to confirm correct placement of the screws and cement.

**Postoperative management:**

Direct postoperative mobilization with pain-adapted full weight-bearing.

**Results:**

Data of 141 patients between January 2018 and August 2022 were analyzed (average age 82 ± 10 years, 89% female). Most of the fractures were type II fragility fractures of the pelvis (FFP; 75%). The median hospital stay was 12 ± 7 days and the median surgery duration for a unilateral SI screw was 26 min. In total 221 S1 screws and 17 S2 screws were applied. No screws showed signs of loosening or migration. Of the five suboptimally placed screws, one screw was removed due to sensory impairment. All patients with cement leakage remained without symptoms.

**Conclusion:**

The surgical technique with the use of a 3D robot-assisted image-guided navigation system is a technique for safe fixation of dorsal fragility fractures of the pelvis and is associated with fewer complications.

**Supplementary Information:**

The online version of this article (10.1007/s00064-024-00871-9) contains supplementary images, explanations and video material, which are available to authorized users.

## Abstract (de)

**Operationsziel:**

Vorstellung und Beschreibung der perkutanen sakroiliakalen Schraubenfixation unter Verwendung eines robotergestützten bildgesteuerten 3D-Navigationssystems und der klinischen Ergebnisse dieser Technik.

**Indikationen:**

Beckenfrakturen mit Beteiligung des hinteren Beckens.

**Kontraindikationen:**

Inoperable Patienten.

**Operationstechnik:**

Planung der Schrauben am diagnostischen Computertomogramm. Registration mit einer Niedrigdosis-Computertomographie (CT) im OP. Laterale Inzision. Kontrolle der K‑Draht-Lage mit den personalisierten Inlet- und Outlet-Aufnahmen. Bei korrekter Position werden die kanülierten Schrauben über den K‑Draht eingebracht. Wir empfehlen eine Augmentation bei Fragilitätsfrakturen. Der Eingriff wird mit einer 3D-CT abgeschlossen, um die korrekte Platzierung der Schrauben und des Zements zu bestätigen.

**Weiterbehandlung:**

Sofortige Mobilisation unter schmerzadaptierter Vollbelastung.

**Ergebnisse:**

Retrospektive Analyse von 141 Patienten (mittleres Alter: 82 ± 10 Jahre, 89 % weiblich), welche zwischen Januar 2018 und August 2022 behandelt wurden. 106 Patienten litten an einer Fragilitätsfraktur des Beckens (FFP) vom Typ II (75 %). Der mediane Krankenhausaufenthalt betrug 12 ± 7 Tage, und die mediane Operationsdauer für eine unilaterale SI-Schraube betrug 26 min. Insgesamt wurden 221 S1-Schrauben und 17 S2-Schrauben eingebracht. Bei der letzten Kontrolle fanden sich keine Anzeichen einer Schraubenlockerung oder -migration. Von den fünf suboptimal platzierten Schrauben wurde eine Schraube aufgrund von Sensibilitätsstörungen entfernt. Alle Patienten mit Zementleckage blieben ohne Symptome.

**Schlussfolgerung:**

Die chirurgische Technik unter Verwendung eines roboterassistierten bildgesteuerten 3D-Navigationssystems erlaubt eine sichere und komplikationsarme Fixation von dorsalen Fragilitätsfrakturen des Beckens.

## Introductory remarks

Percutaneous screw osteosynthesis has emerged as the procedure of choice for fixing the posterior pelvic ring due to its minimally invasive nature [18], lower risk of infection [4], rapid recovery [19] precise screw placement [14], and cost-effectiveness. First described in 1995 by Routt, this technique was initially challenging with high complication rates, mainly due to screw misplacement, nerve injuries, or screw failure (0–33%) [18]. But, over the years, this technique has improved, and complication rates are decreasing [7].

One of the most challenging aspects of the percutaneous screw fixation is intraoperative imaging [9]. Weak contrast due to decreased bone density, bowl gas overlay, and symphysis superposition can limit imaging quality [12]. Implementing a 3D robot-assisted image-guided navigation system might provide a reliable solution. By calibrating the 3D scan prior to surgery, the surgeon can easily switch between personalized inlet, outlet, and lateral views of the pelvis, ensuring consistency and efficiency throughout the procedure. Moreover, 3D robot-assisted image-guided navigation system allows for precise planning and intraoperative guidance of screw placement. Furthermore, intraoperative 3D scans can be obtained to confirm correct screw positions and allow for immediate correction of any misplacements.

While the advantages of this 3D robot-assisted image-guided navigation technology extend to all pelvic fractures, we have chosen to focus on its application in fragility fractures of the pelvis (FFP) due to the increased benefits it provides to this frail patient population.

The paper aims to present a standardized surgical technique and to address technical challenges and possible solutions. We share our clinical experience with 3D robot-assisted image-guidance in the surgical treatment of FFP using percutaneous sacroiliac screws.

## Surgical principle and objective

The objective of percutaneous sacroiliac screw osteosynthesis of the pelvis is to achieve stabilization with minimal soft tissue dissection and short operation time. The use of a robot-assisted 3D image-guided navigation system allows for precise execution of the preoperative plan by intraoperative navigation of the screw path with the help of individually definable and exactly reproducible imaging planes by means of a high-resolution 3D scan.

## Advantages

- Short operation duration [15]
- Minimal blood loss
- Limited soft-tissue dissection
- Precise determination, guidance, and control of the screw position
- Early patient mobilization especially in frail patients
- Reduced technical limitations in obese patients
- Providing maximal contrasted x‑ray in osteopenic patients or bowl gas overlay

## Disadvantages

- Requires access to a hybrid operating room
- Patient movement during the procedure may lead to loss of matching with the navigation system
- Limited use of needle guidance in the anterior pelvic ring due to potential movement during posterior screw insertion and greater distance to the table
- Learning curve for handling the hybrid operating room
- Longer preparation, including draping and matching 3D scan

## Indications

- Fragility fractures of the pelvis type II, III, and IV
- High-energy pelvic fractures

## Contraindications

- The patient is not fit for surgery according to anesthesiology assessment
- Infection at site of screw insertion

## Patient information

- General risks of surgery
- Screw loosening over time
- Injury to the nerves (nerve roots S1, S2, and L5)
- Injury to the vessels (branches of the superior gluteal artery)

## Preoperative work-up

**Fig. 1:**
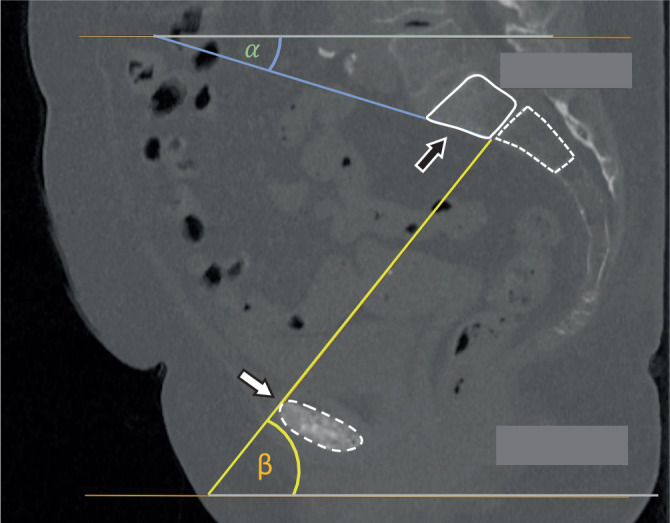
Determination of the personalized inlet angle (*α*) and personalized outlet angle (*β*) in the sagittal view. The dashed line encircles the bony part of the symphysis, the solid line encircles the sacral vertebral bodies, the black arrow points to the S1 body

- Perform a clinical examination of the pelvis, focusing on compression pain and tenderness over the sacrum and pubic rami.
- Conduct a clinical neurological examination of the lower extremities.
- Utilize a diagnostic CT for detection and classification of the fracture (appendix 1). Conventional X‑rays carry a high risk of misclassification [2].
- Employ the diagnostic CT to measure the angles for the personalized inlet and outlet view, which are critical aspects of the presented method (Fig. 1). A comprehensive explanation of the personalized inlet and outlet angles is illustrated in appendix 2

## Instruments

- Standard surgical instruments for osteosynthesis (scalpel, long pean, light mallet, screwdriver, power drill with guidewire adapter, forceps, needle holder)
- Long 2.8 mm guidewires
- Changing canula with hexagonal ending for secure insertion into the screw head
- 7.3 mm cannulated screws with washer
- If cement augmentation is considered: 7.5 mm fully threaded, cannulated, and fenestrated screws with integrated washer and augmentation kit with slow polymerizing polymethyl methacrylate (PMMA) bone cement
- Optional: non-cannulated 3.5 or 4.5 mm or cannulated 7.3 mm fully threaded screws

## Anesthesia and positioning

**Fig. 2:**
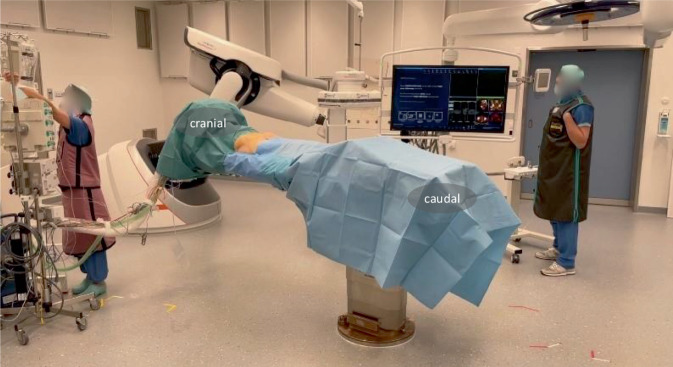
Positioning and draping of the patient. On two movable screens customizable views with intra- and preoperative images may be viewed. The robotic C‑arm is controlled by an operator or the control panel may be draped and operated by the surgeons. For the acquisition of a 3D scan the situs is additionally covered with loose drape

- General anesthesia or compliant patient with spinal anesthesia
- All patients receive a perioperative antibiotic prophylaxis (cefazolin 2 g single shot)
- Supine positioning allows for fixation of the anterior and posterior pelvic ring and reduces anesthesiologic challenges. Prone positioning is also possible if the anterior pelvic ring is addressed in a retrograde manner.
- The patient should be placed in the center of a radiolucent table, pelvic tilt and/or rotation should be avoided
- Arms are positioned in front of the patients face and positioned in an arm holder device
- Disinfection area includes the visible abdomen from the navel to mid thighs. Draping consists of a drape deeply tucked beneath the patient on either side, a self-adherent U‑drape starting just above the confluence of the major labia or the base of the penis, and a self-adherent drape covering half the navel and above. The loose ends of the drapes are collated to the inferior part of the radiolucent table to allow for free movement of the C‑arm (Fig. 2). After definitive patient positioning and draping, it must be tested that the C‑arm can move freely around the patient.
- It is important that the patient lays completely still, and no position adjustments are made after the 3D scan.

## Surgical technique

(Figs. 3, 4, 5, 6, 7, 8, 9, 10 and 11)

**Fig. 3:**
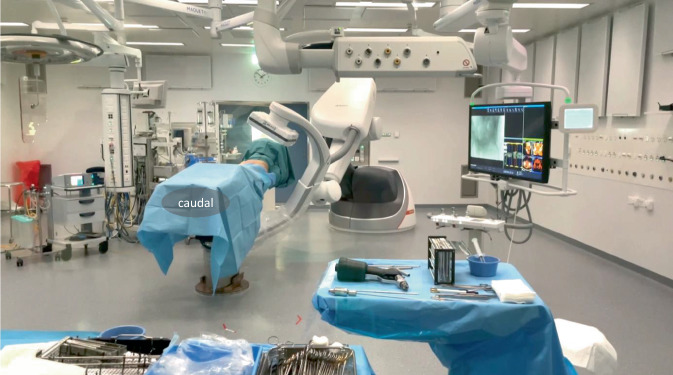
The first step is performing a 3D scan for matching with the diagnostic CT scan for abbreviations and further explanations see *appendix* *3*. The staff leaves the room during acquisition of the 3D scan to reduce radiation exposure. Matching consists of manual approximation, but final matching is done by the computer

**Fig. 4:**
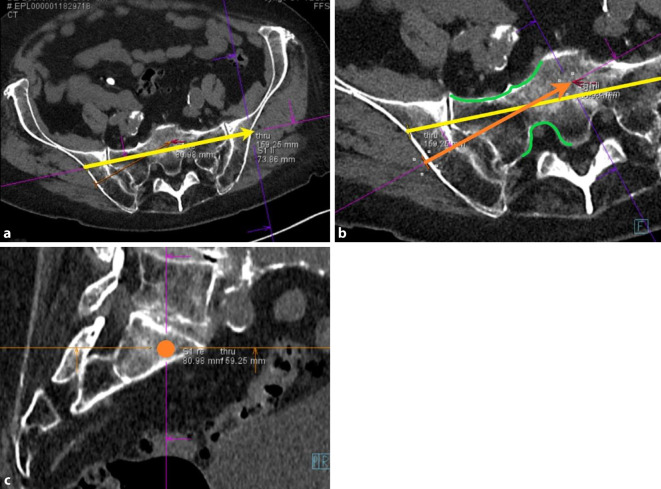
To facilitate an efficient workflow, screw pathway planning on the diagnostic CT is performed while preparing the patient in the hybrid theatre. The process of planning the screw pathways involves several steps. Firstly, ensure symmetrical alignment of the pelvis in all three planes. Then, an auxiliary pathway is defined (yellow arrow*,* **a**), to achieve the lateral view intraoperatively (Fig. 5) which is essential to determine the optimal entry point for the SI screw. The predefined personalized inlet, outlet, and lateral views (see *appendix* *2*) are saved by the software system to allow swift intraoperative transition between these views. The next step involves planning the desired screws in the coronal and axial slices (*orange arrow* and *dot*, ** b**, **c**). Two critical factors to consider are: ensuring the entry point is not too dorsal to avoid conflict with the operating table due to supine positioning of the patient, and the planned screw ideally transversing the corridor in the S1 body centrally between the ventral sacral cortex and the neuroforamen (green lines, **b**) [5]. The correct intraosseous path can be checked by scrolling forward and backward along the planned screws in the sagittal plane. These planned screw pathways can be projected with dotted lines on all fluoroscopic views intraoperatively (as shown in Figs. 6, 7, and 9)

**Fig. 5:**
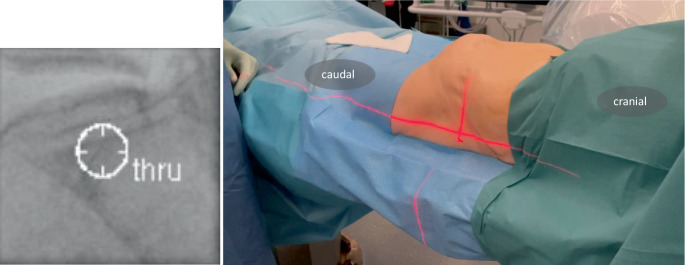
The surgeon stands on the ipsilateral side. Following team time-out, the C‑arm is positioned in the predefined lateral view (yellow arrow, Fig. 4a). The robotic C‑arm software enables approach to a planned screw path in the so-called bull’s eye view, aligning the central X‑ray beam precisely with the screw path. In Fig. 5, this path is displayed in the bull’s eye view as “thru”. To gain more space to work between the patient and the C‑arm, the C‑arm must be moved to the ipsilateral side. Typically, this results in a parallax effect if the patient is not aligned to the table. Manually this may be corrected by moving the C‑arm so the center of the image is aligned with planned entry point. The laser on the patient’s skin indicates the center of the fluoroscopy image. With SI screws typically positioned in a posterior-to-anterior direction (approximately 10–20°), the entry point is more dorsal, requiring a skin incision made 1–2 cm dorsal to the laser marker

**Fig. 6:**
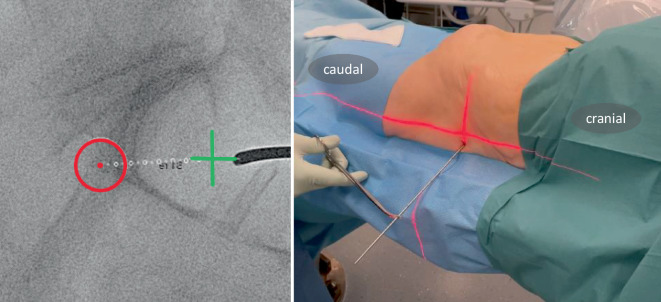
Lateral fluoroscopic image with real-time projection of the planned guide-wire entry point (green cross), its direction (dotted line), and endpoint (red circle). The guidewire is positioned on the bone. Once correctly positioned, it is introduced into the bone via light mallet strikes until it penetrates three cortices. If the correct angle in the axial plane is achieved (usually 10–20°), the guidewire advances along the planned trajectory in the lateral view. The planned screw pathway is visible at all times on the fluoroscopic images, to allow for navigation and direct adjustment of the guidewire. To minimize the surgeon’s radiation exposure, the guidewire is held with a forceps

**Fig. 7:**
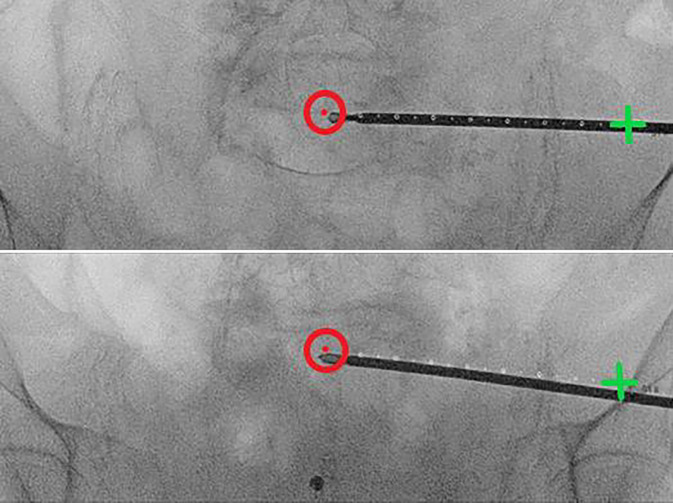
The guidewire position is verified using the predefined personalized inlet and outlet angles (see *appendix* *2*). While alternating between these personalized angles, the guidewire can be repositioned and driven into its final position with the drill. Key to this process is keeping in mind the vital landmark: the slightly S‑shaped anterior cortex of the S1 vertebral body. In uncertain scenarios, a higher dosage x‑ray may be acquired, or the contralateral side extrapolated for reference. Possible mismatch between diagnostic and intraoperative CT may result in an inaccurate planned screw pathway overlay; in such cases, the overlay can still provide an estimated guidewire pathway and end position. After final placement of the guidewire, its length is measured

**Fig. 8:**
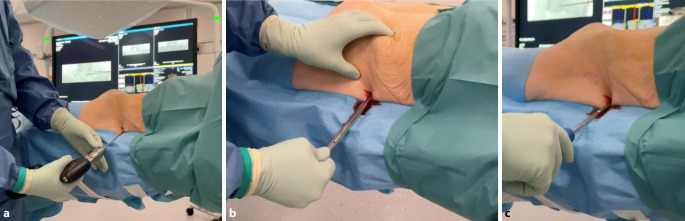
**a** Measurement of the intended screw length. **b** The guidewire is over drilled through three cortices with a 5.0 mm drill into the sacrum. Due to the osteoporotic bone, it is not necessary to over-drill the entire length of the guidewire. **c** A cannulated screw is manually inserted over the guidewire; a washer is utilized to prevent intrusion into the ilium. Correct snug positioning of the washer on the cortex is checked with an x‑ray in 20° ipsilateral tilt

**Fig. 9:**
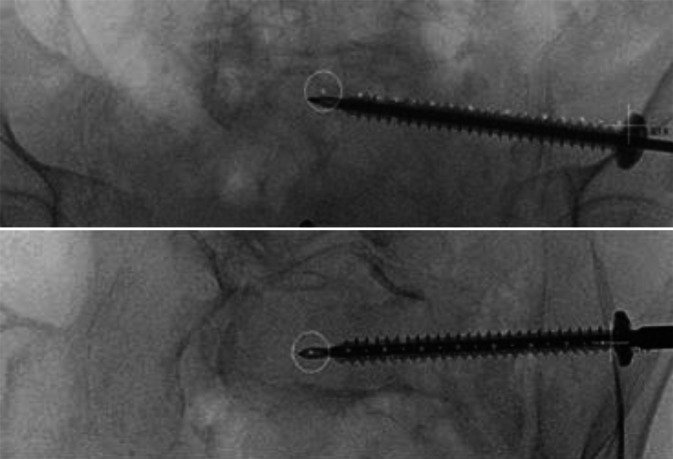
At this stage, a 3D scan can be performed to verify the screws’ correct positioning. In our department, 3D scans are typically conducted at the end of the procedure. For introducing a second screw on the contralateral side, a mirrored version of the original lateral view is used to identify the correct entry point

**Fig. 10:**
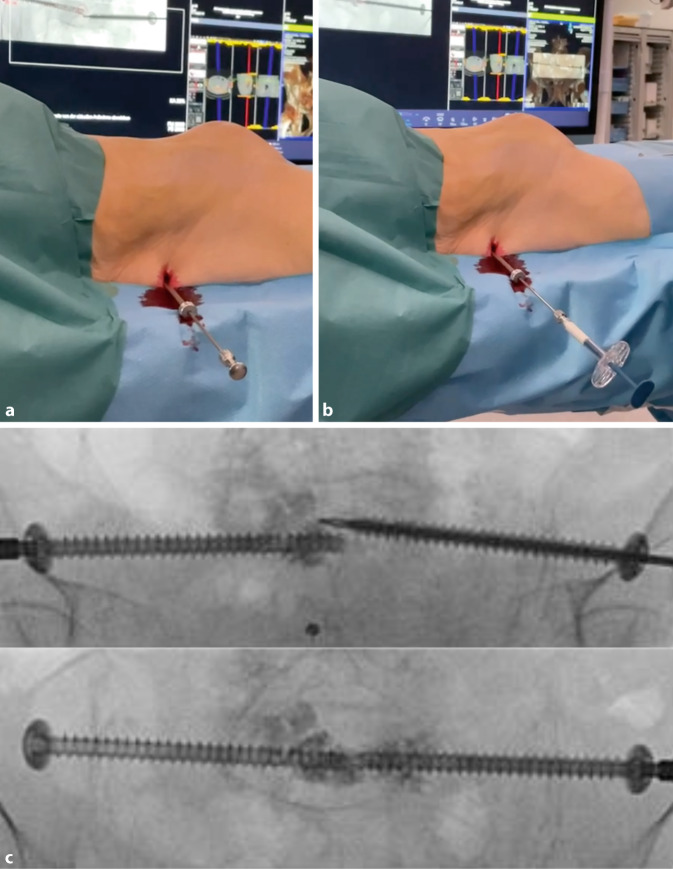
For FFP we recommend augmentation [4, 8, 13]. **a** and **b** The augmentation trocar is placed over the guidewire and introduced into the screw head. The cement is prepared as per manufacturer guidelines, and the guidewire is replaced with the augmentation cannula before connecting a syringe filled with cement. **c** The cement typically spreads around the screw tip and via the fenestrations, with around 1–2 ml of cement used per screw. If the distribution is atypical, cement augmentation should be aborted

**Fig. 11:**
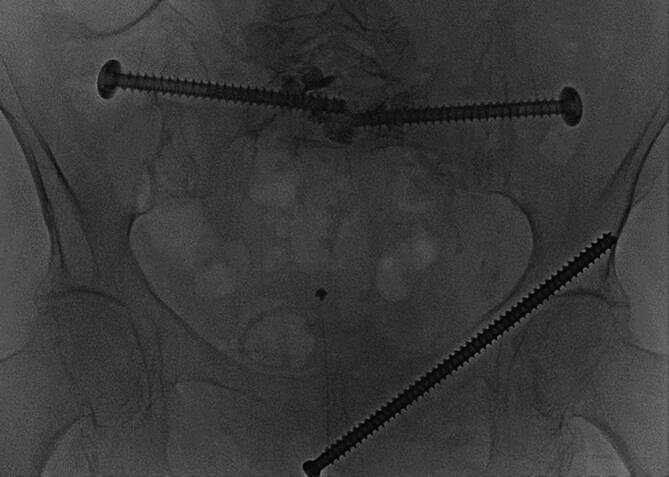
An overview of the pelvis showcasing the final results. All incisions are irrigated and closed by suture

## Special surgical considerations

- For standardized positioning and collision-free movement, we recommend floor markings for all mobile devices, such as infusion stand, ventilator machine or suction machine.
- The described sacroiliac screws and retrograde superior ramus screw may be combined with transiliosacral screws, sacral bars or sacroiliac screws in the S2 body.
- This technique may be combined with open procedures and is highly adaptable.
- The typical personalized outlet angle is approximately 40°. If this angle is not achievable intraoperatively due to potential collision of the C‑arm with the table, the highest possible angle should be accepted. The procedure becomes less complex with an increased difference between the angels of the personalized inlet and outlet views. Ideally, this difference would be 90°, as changes in one plane do not necessarily result in changes in the other.
- However, typically the difference between angles is significantly lower than 90° (also see *appendix 2*).
- The personalized inlet and outlet angle, described in *appendix 2*, are especially of advantage in elderly patients with dysmorphic pelvic characteristics [11].
- In older patients with more elastic skin and soft tissues, guidewire repositioning is relatively easy compared to younger patients with firm skin, who may require pullback beneath the skin for redirection. Penetration of the washer into the ilium should be avoided because it typically causes persisting pain.
- Even in cases in which sacral fractures are not evident on CT but patients report pain on sacral palpation, an imminent fracture may be assumed and, thus, a screw may be inserted due to low complication risk and minimal additional time (Table 1; [1])

**Table 1:** Our approach for screw placement indication

	Painful	Not painful
Fracture visible in CT	+	+
Fracture not visible in CT	+	–

+ means screw indicated, − means no screw indicated

## Postoperative management

- Direct postoperative mobilization with pain adapted full weight bearing
- Daily physiotherapy focused on stability, gait security and prevention of falling, breathing exercises
- Main goal should be to regain the preoperative mobility and independence level and return to same domicile as before the fracture
- Thromboembolic prophylaxis for at least 6 weeks postoperative [6]
- Removal of skin sutures after 2 weeks
- Clinical and radiological (anteroposterior pelvis X‑ray) follow-up after 6 weeks and 3–4 months
- In cases of persisting pain until 6 weeks or pathological findings on conventional x‑ray additional CT is recommended

## Errors and complications

- Screw misplacement in the spinal canal or the neuroforamen with contact to the nerve root of S1 or S2
- Cement leakage posterior into the neuroforamen or spinal canal with possible neurological symptoms
- Intraoperative or postoperative bleeding, for example, injury of the superior gluteal artery. If recognized intraoperatively, it may be controlled by image-guided embolization.
- General surgical and medical complications

## Results

**Fig. 12:**
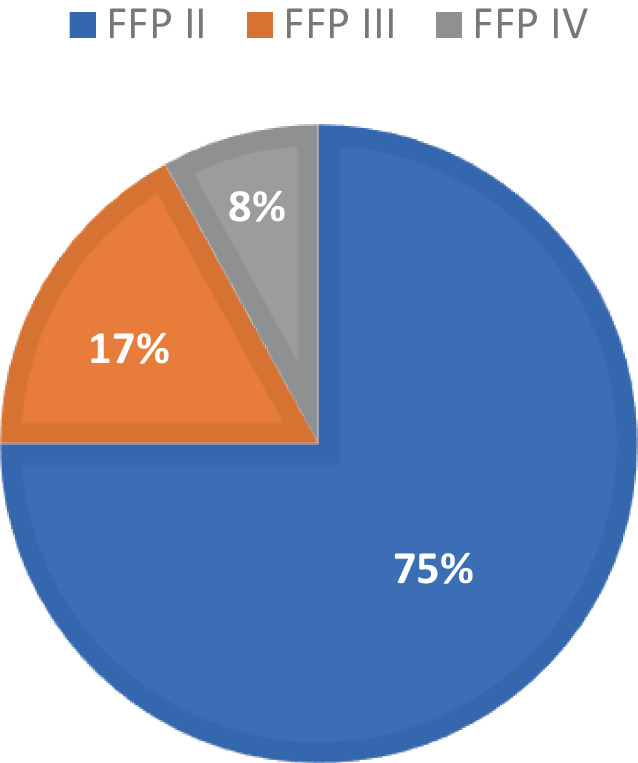
Distribution of fragility fractures of the pelvis (FFP) types

Data were collected from 141 patients who underwent 3D robot-assisted image-guided percutaneous sacroiliac screw fixation for fragility fractures between January 2018 and August 2022. The average patient age was 82 ± 10 years (95% confidence interval 78–90), with a majority being female (89%). Most of the fractures were type II, consistent with the literature (Fig. 12; [16, 17]).

Table 2 summarizes patient characteristics, highlighting gender distribution, median age, hospital stay duration, and timeline from trauma to diagnosis and surgery.

**Table 2:** Patient characteristics

	*n*		95% CI
*Female/Male (%)*	126/15	89%/11%	–
*Age (median in years)*	–	85	78–90
*Length of hospital stay (median in days)*	–	10	7–15
*Time from trauma till diagnosis (median in days)*	–	1	0–14
*Time from trauma till surgery (median in days)*	–	13	6–28
*Time from first diagnosis until surgery (median in days)*	–	6	4–13

The time from trauma to surgery varied significantly due to referral delays or failure of conservative management. On average, diagnoses were made on the first day following trauma, and surgeries were typically performed 6 days postdiagnosis. As expected, surgery duration increased with the number of screws placed, with the median duration for one sacroiliac screw placement being 26 min (Table 3).

**Table 3:** Surgery duration

	*n*		Range
*Surgery duration (median in minutes)*	–	70	12–272
Unilateral SI screw (median in minutes)	6	26	12–35
Bilateral SI screws (median in minutes)	16	49	34–102
SI + Transiliosacral screws (median in minutes)	1	179	–
Unilateral SI + anterior screw (median in minutes)	48	63	28–247
Bilateral SI + anterior screw (median in minutes)	63	75	47–272
Combination all (median in minutes)	3	154	79–157

Table 4 offers a detailed breakdown of the 238 screws placed in 141 patients, outlining the type of screws used and complications encountered. Direct postoperative complications, such are screw misplacement, cement leakage, bleeding or postoperative infection are reported for all patients. Long-term complications, such as secondary screw migration, is reported for 112 patients. The mean follow-up of those patients was 22 weeks (range 4–184 weeks). Five sacroiliac screws (1.3%) were placed suboptimally, identified by a 3D scan performed at the end of the procedure. These placements were within acceptable limits and only one required removal 9 days postoperatively due to sensory impairment. Five screws exhibited cement leakage into the neuroforamina. However, none of these patients reported neurological symptoms.

**Table 4:** Distribution of screws in numbers and % of all patients

	S1 (*n* = 221)	S2 (*n* = 17)
*Left*	27 (7.0%)	8 (2.1%)
*Right*	26 (6.8%)	7 (1.8%)
*Both*	84 (21.9%)	1 (0.3%)
*Complications*		
Screw misplacement ventral	4 (1.0%)	0
Screw misplacement foramen	0	1 (0.3%)
Cement leakage (foramen or spinal canal)	5 (1.3%)	0
Cement leakage (other)	5 (1.3%)	1 (0.3%)
Infection	0	0
Secondary screw migration	0	0

Given the low incidence and minimal clinical impact of screw misplacement and cement leakage, it has become common practice in our hospital to perform a 3D scan at the end of the procedure, following augmentation. However, a 3D scan can be carried out at any point during surgery if there is uncertainty about correct screw placement depending on local preferences and level of expertise.

No loosening or migration was observed in sacroiliac screws. There were no postoperative infections. Intraoperative bleeding was reported in 2 patients: one was controlled by compression and in the other patient an angiography was performed showing that the bleeding had stopped spontaneously.

The percutaneous screw fixation technique for pelvic fragility fractures has evolved over time, addressing various technical challenges. While technological advancements from 2D to 3D and nonnavigated to navigated screw placement did not significantly reduce complication rates, it positively influenced screw misplacement rates and operation duration [3, 10, 15, 20].

## Supplementary Information

Appendix 1: simplification of the classification of FFP according to Rommens

Appendix 2: personalized inlet and outlet angle

Appendix 3: Abbreviations and dictionary

Video

## Declarations

For this article no studies with human participants or animals were performed by any of the authors. All studies mentioned were in accordance with the ethical standards indicated in each case.
