# Supplementary material for: Percutaneous sacroiliac screw fixation with a 3D robot-assisted image-guided navigation system: Technical solutions
Source: Oper Orthop Traumatol. 2024 Nov 18;37(1):3–13. doi: 10.1007/s00064-024-00871-9 (PMC11790701; doi:10.1007/s00064-024-00871-9)
Supplement: Supplementary file 1 — Appendix 1: simplification of the classification of FFP according to Rommens [file 64_2024_871_MOESM1_ESM.docx]

**Appendix 1**

| **Simplification of the classification of FFP according to Rommens** | |
| --- | --- |
| 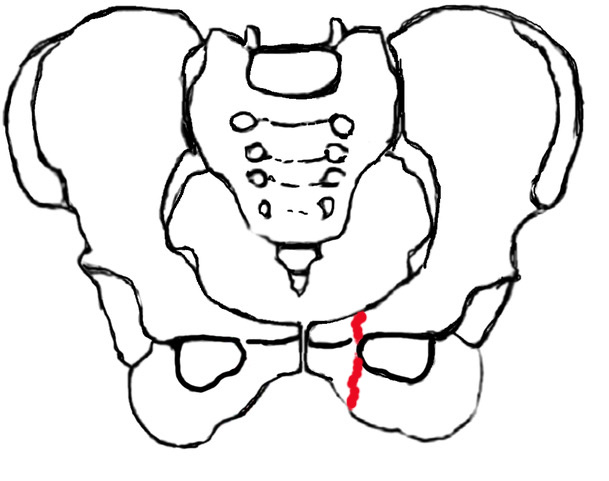 | 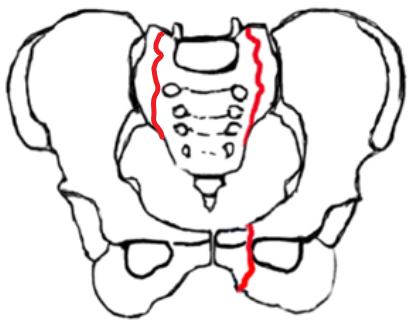 |
| Typ I  Anterior | Typ II  Uni-/bilateral posterior undislocated |
| 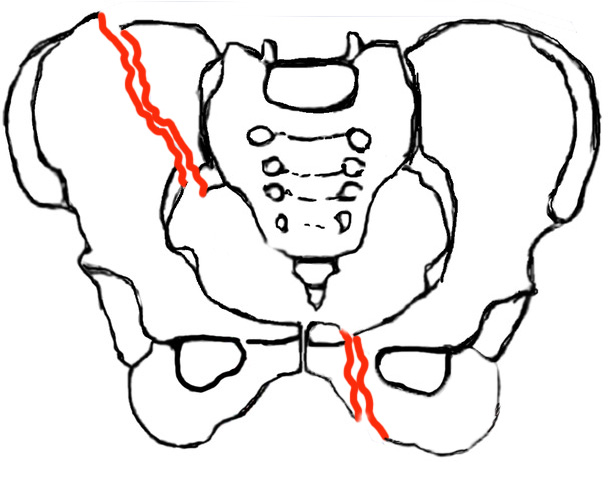 | 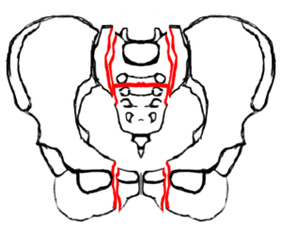 |
| Typ III  Unilateral posterior dislocated | Typ IV  Bilateral posterior dislocated |
