# Supplementary material for: Percutaneous sacroiliac screw fixation with a 3D robot-assisted image-guided navigation system: Technical solutions
Source: Oper Orthop Traumatol. 2024 Nov 18;37(1):3–13. doi: 10.1007/s00064-024-00871-9 (PMC11790701; doi:10.1007/s00064-024-00871-9)
Supplement: Supplementary file 2 — Appendix 2: personalized inlet and outlet angle [file 64_2024_871_MOESM2_ESM.pptx]

## Slide 1
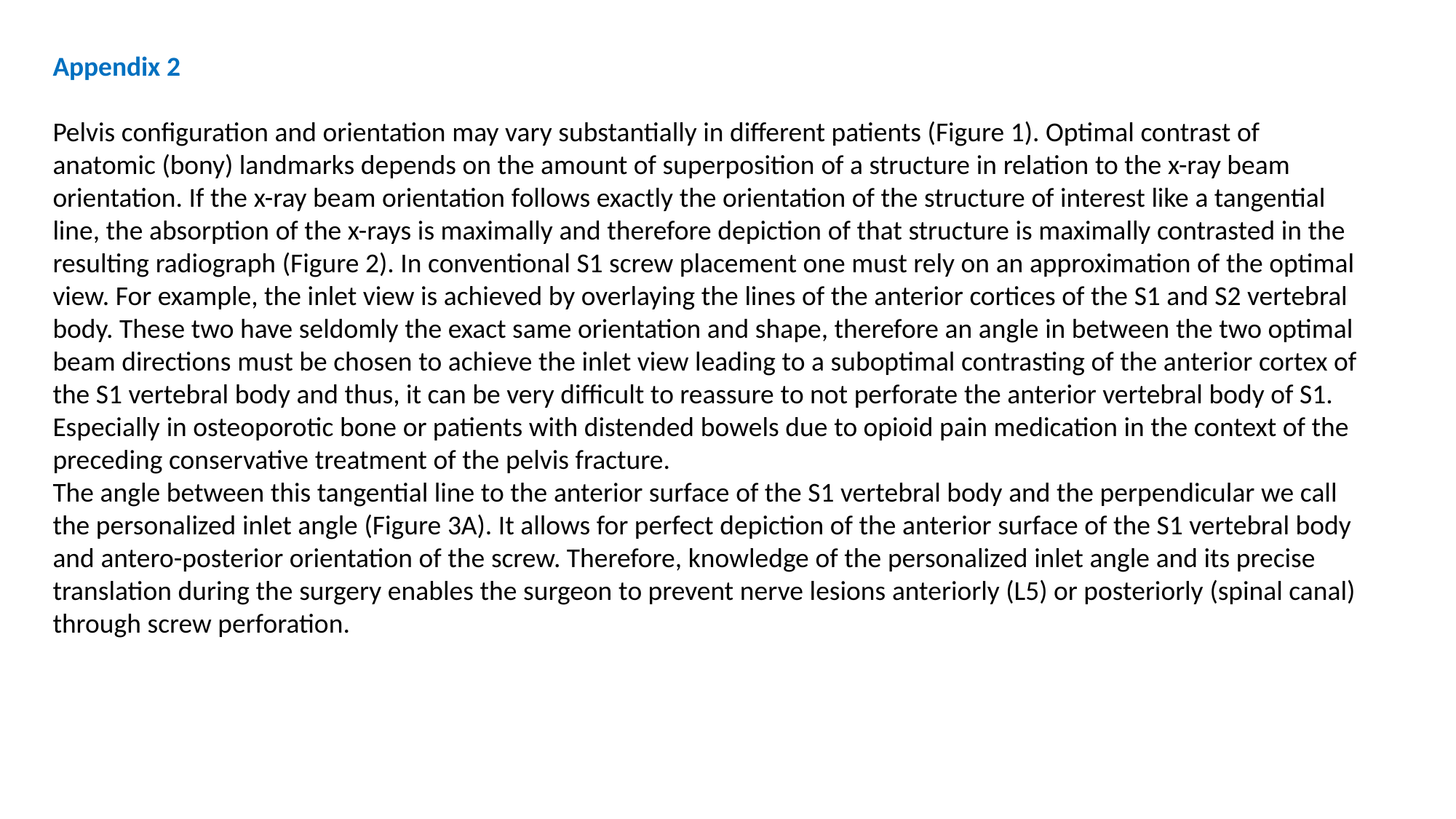

Appendix 2
Pelvis configuration and orientation may vary substantially in different patients (Figure 1). Optimal contrast of anatomic (bony) landmarks depends on the amount of superposition of a structure in relation to the x-ray beam orientation. If the x-ray beam orientation follows exactly the orientation of the structure of interest like a tangential line, the absorption of the x-rays is maximally and therefore depiction of that structure is maximally contrasted in the resulting radiograph (Figure 2). In conventional S1 screw placement one must rely on an approximation of the optimal view. For example, the inlet view is achieved by overlaying the lines of the anterior cortices of the S1 and S2 vertebral body. These two have seldomly the exact same orientation and shape, therefore an angle in between the two optimal beam directions must be chosen to achieve the inlet view leading to a suboptimal contrasting of the anterior cortex of the S1 vertebral body and thus, it can be very difficult to reassure to not perforate the anterior vertebral body of S1. Especially in osteoporotic bone or patients with distended bowels due to opioid pain medication in the context of the preceding conservative treatment of the pelvis fracture.
The angle between this tangential line to the anterior surface of the S1 vertebral body and the perpendicular we call the personalized inlet angle (Figure 3A). It allows for perfect depiction of the anterior surface of the S1 vertebral body and antero-posterior orientation of the screw. Therefore, knowledge of the personalized inlet angle and its precise translation during the surgery enables the surgeon to prevent nerve lesions anteriorly (L5) or posteriorly (spinal canal) through screw perforation.

## Slide 2
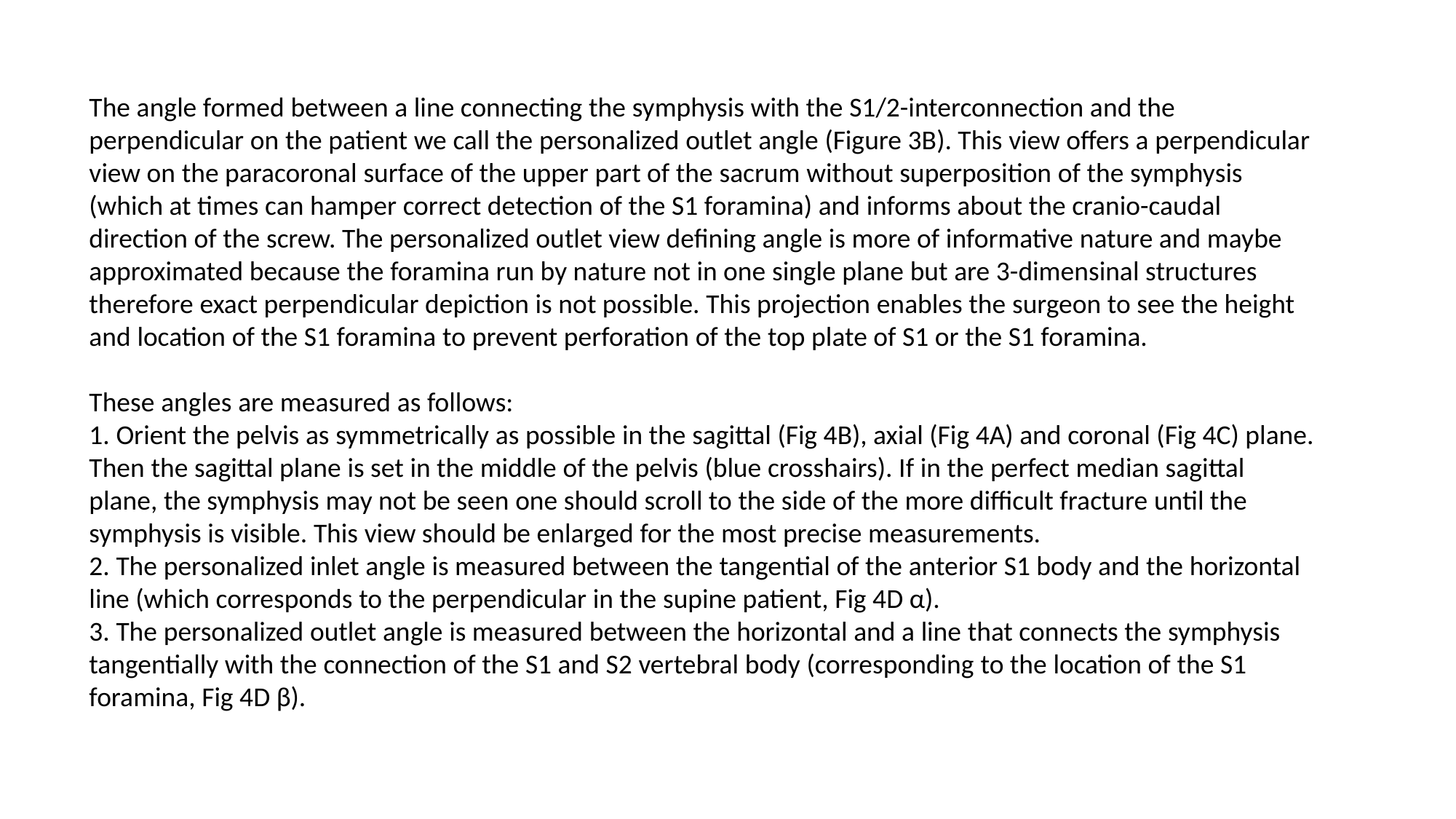

The angle formed between a line connecting the symphysis with the S1/2-interconnection and the perpendicular on the patient we call the personalized outlet angle (Figure 3B). This view offers a perpendicular view on the paracoronal surface of the upper part of the sacrum without superposition of the symphysis (which at times can hamper correct detection of the S1 foramina) and informs about the cranio-caudal direction of the screw. The personalized outlet view defining angle is more of informative nature and maybe approximated because the foramina run by nature not in one single plane but are 3-dimensinal structures therefore exact perpendicular depiction is not possible. This projection enables the surgeon to see the height and location of the S1 foramina to prevent perforation of the top plate of S1 or the S1 foramina.
These angles are measured as follows:
1. Orient the pelvis as symmetrically as possible in the sagittal (Fig 4B), axial (Fig 4A) and coronal (Fig 4C) plane. Then the sagittal plane is set in the middle of the pelvis (blue crosshairs). If in the perfect median sagittal plane, the symphysis may not be seen one should scroll to the side of the more difficult fracture until the symphysis is visible. This view should be enlarged for the most precise measurements.
2. The personalized inlet angle is measured between the tangential of the anterior S1 body and the horizontal line (which corresponds to the perpendicular in the supine patient, Fig 4D α).
3. The personalized outlet angle is measured between the horizontal and a line that connects the symphysis tangentially with the connection of the S1 and S2 vertebral body (corresponding to the location of the S1 foramina, Fig 4D β).

## Slide 3
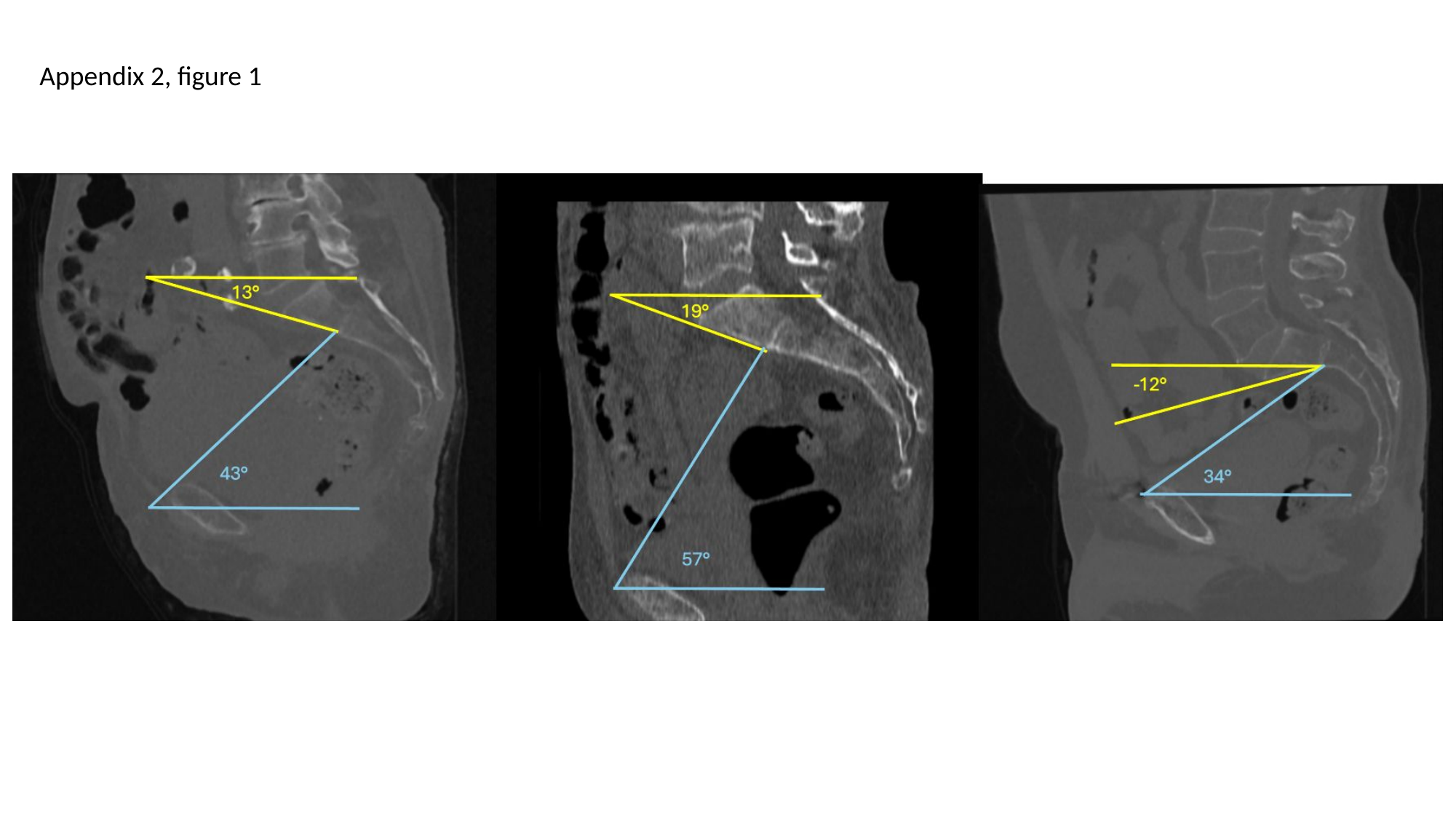

Appendix 2, figure 1

## Slide 4
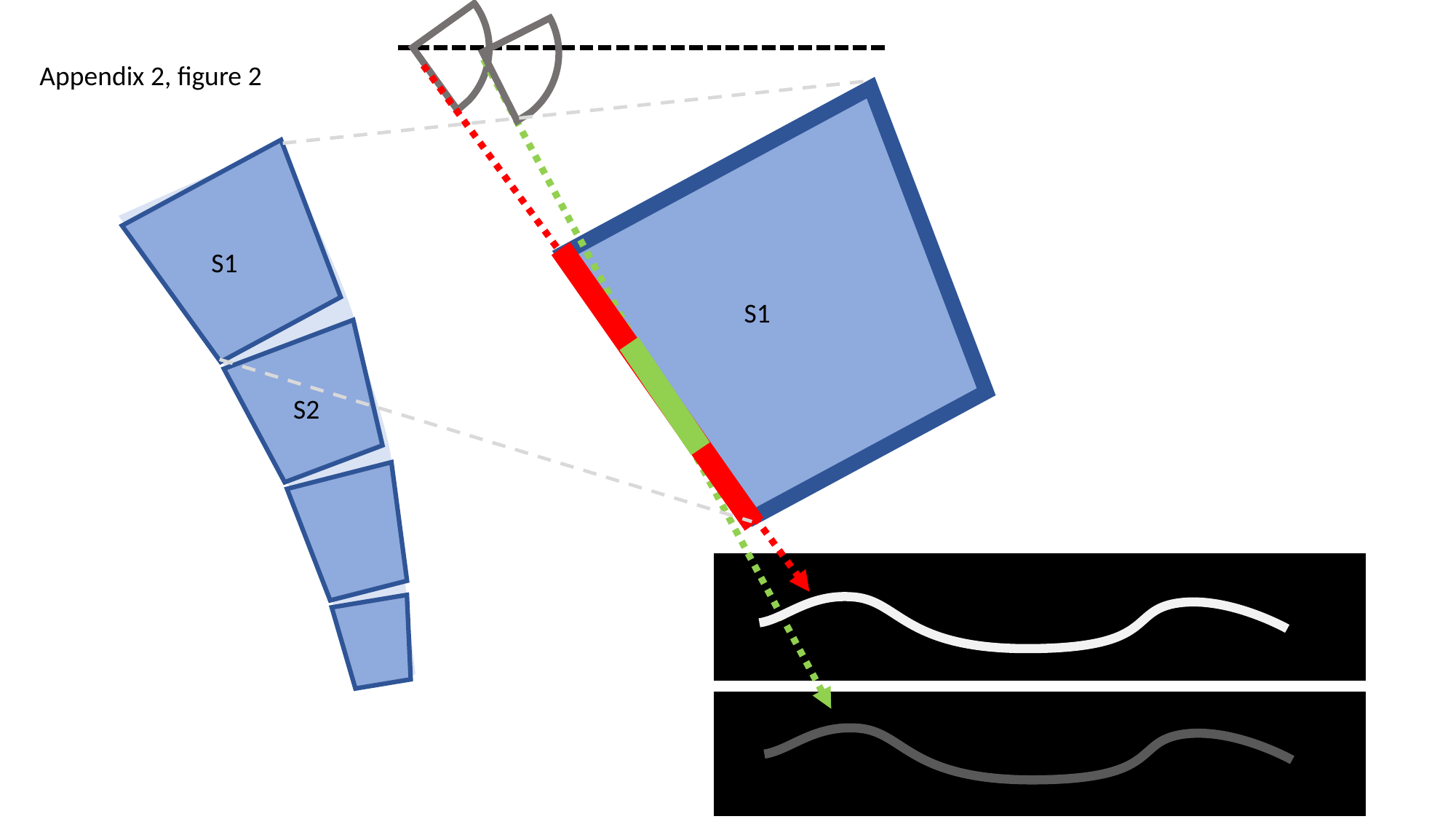

Appendix 2, figure 2
S1
S1
S2

## Slide 5
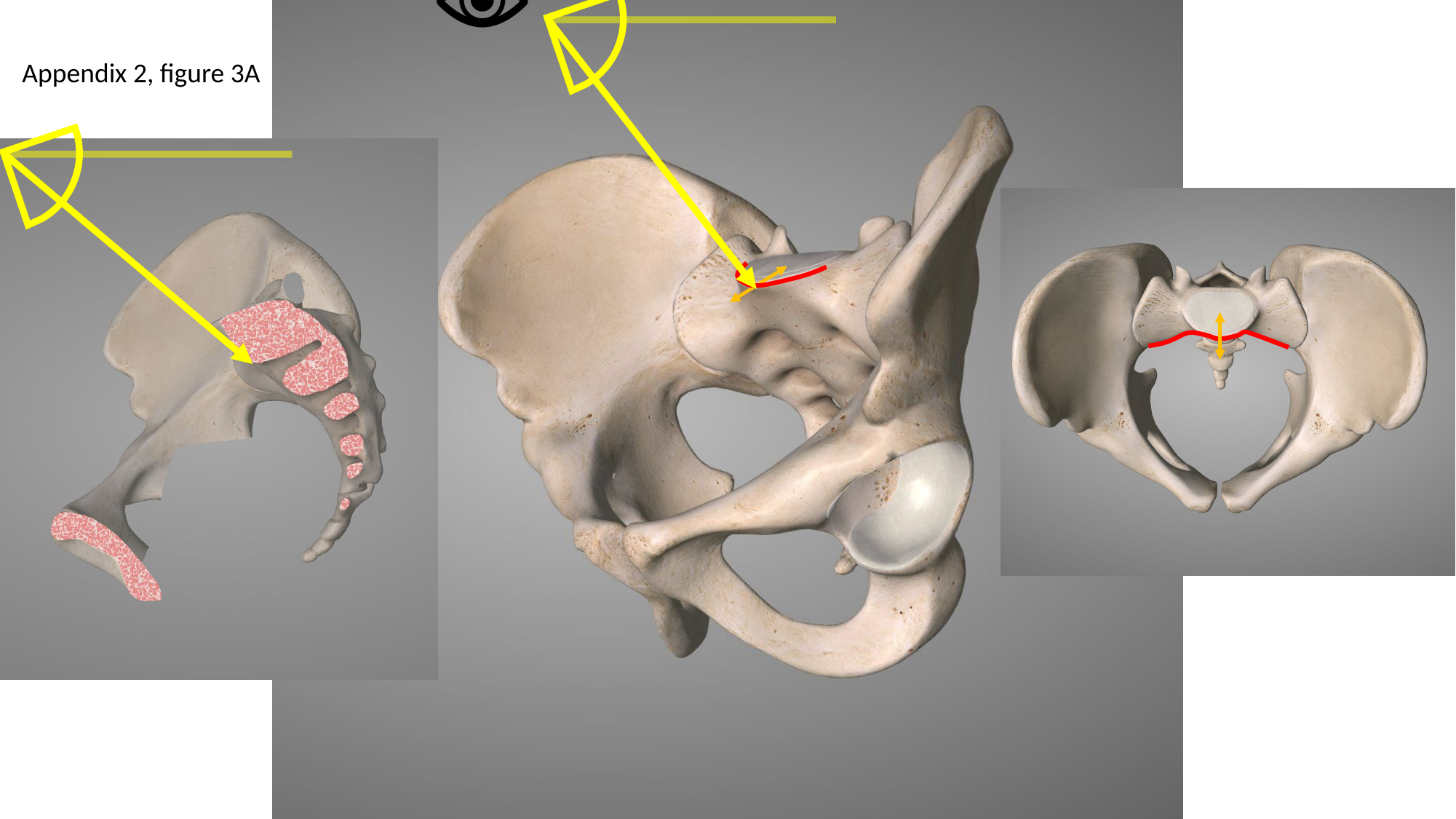

Appendix 2, figure 3A

## Slide 6
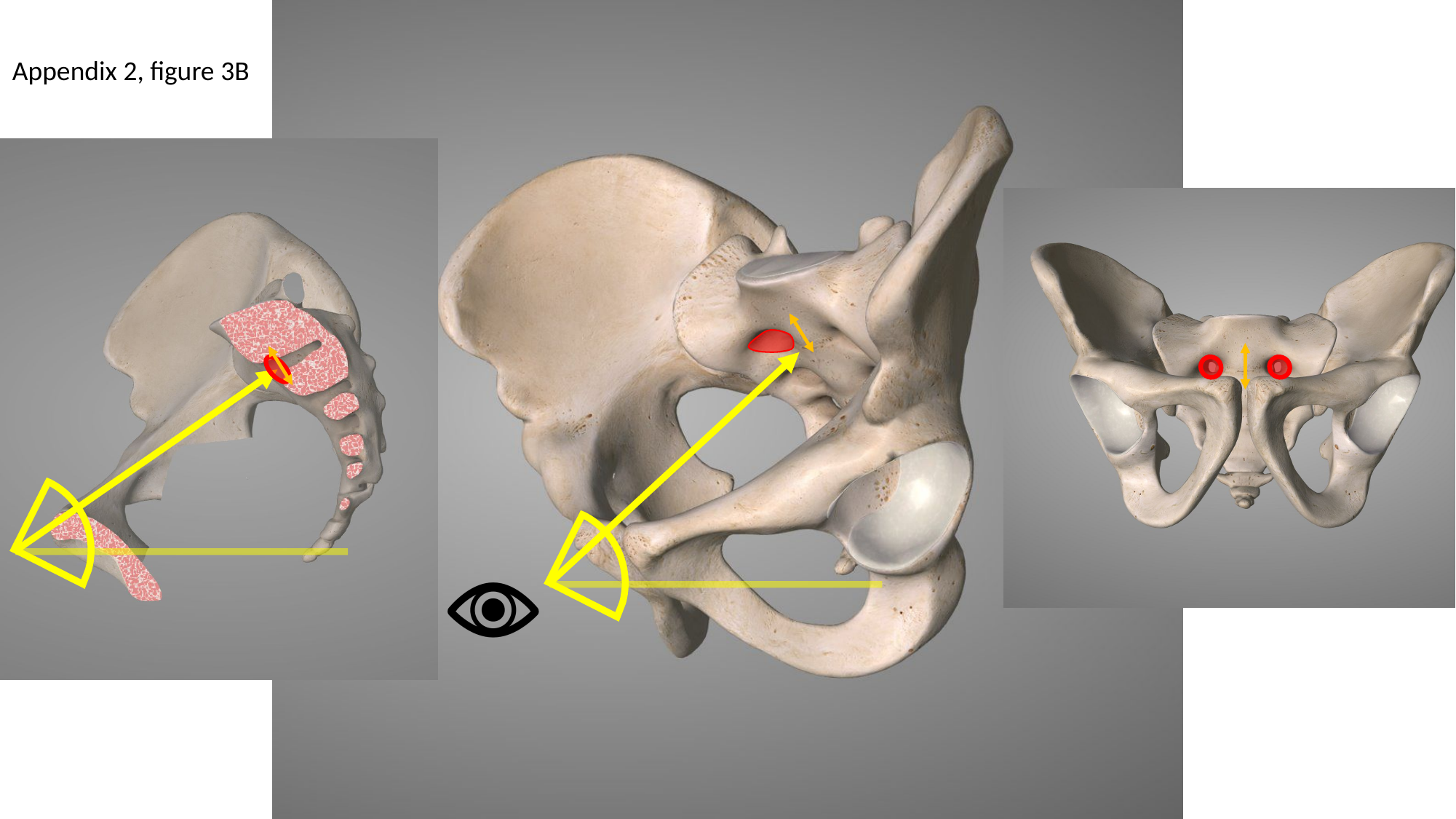

Appendix 2, figure 3B

## Slide 7
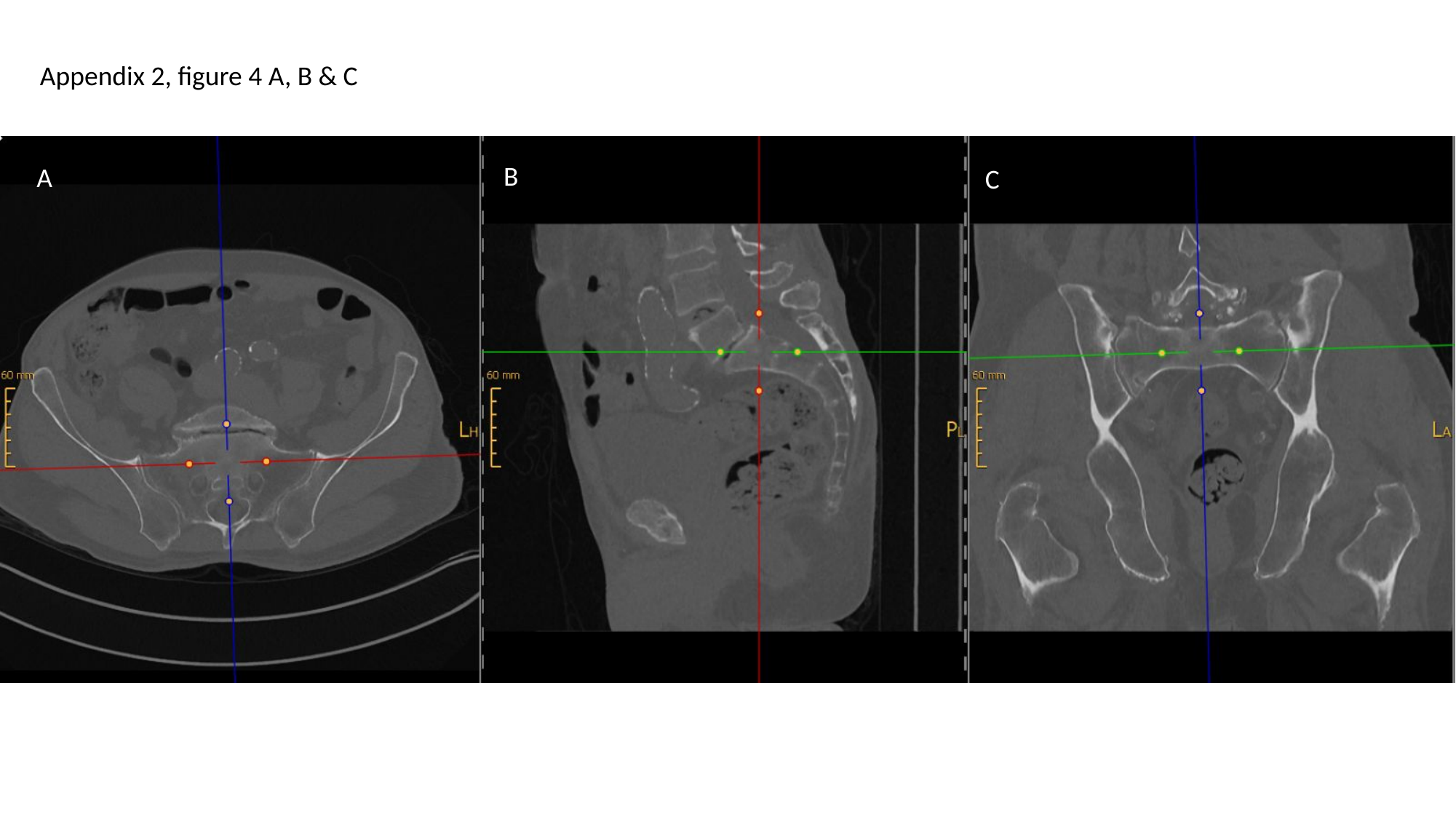

Appendix 2, figure 4 A, B & C
B
A
C

## Slide 8
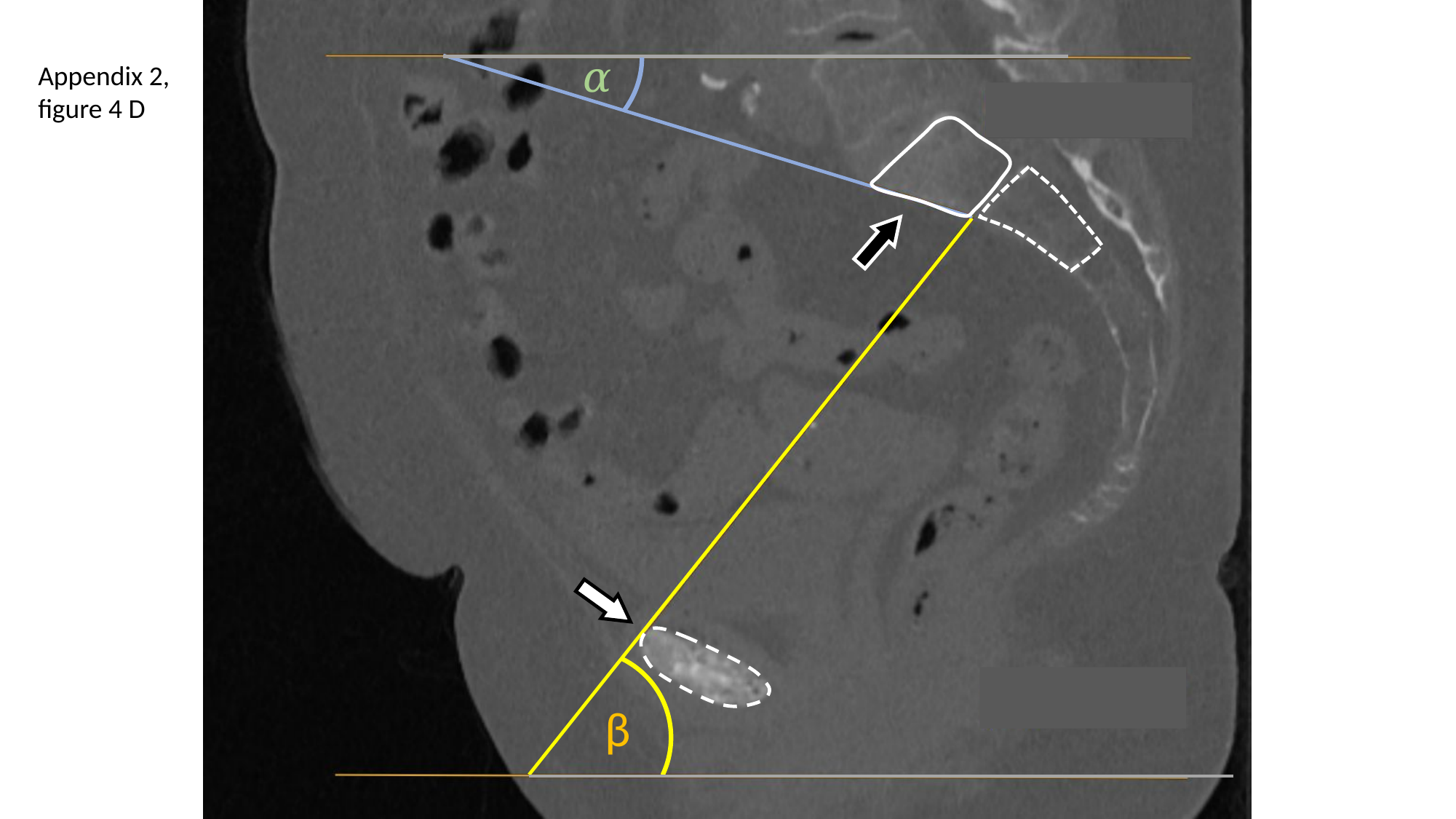

𝛼
Appendix 2, figure 4 D
β
