# Supplementary material for: Percutaneous sacroiliac screw fixation with a 3D robot-assisted image-guided navigation system: Technical solutions
Source: Oper Orthop Traumatol. 2024 Nov 18;37(1):3–13. doi: 10.1007/s00064-024-00871-9 (PMC11790701; doi:10.1007/s00064-024-00871-9)
Supplement: Supplementary file 3 — Appendix 3: Abbreviations and dictionary [file 64_2024_871_MOESM3_ESM.docx]

**Appendix 3**

Abbreviations and dictionary

| FFP | Fragility fracture of the pelvis |
| --- | --- |
| SI | Sacroiliac |
| LUKS | Cantonal hospital of Lucerne (Luzerner Kantonsspital) |
| Personalized inlet angle | Depiction of the anterior cortex (paracoronal surface) of the S1 vertebral body |
| Personalized outlet angle | Unhindered and perpendicular depiction of the paracoronal surface of the upper part of the sacrum without superposition of the symphysis |
|  |  |
| **Types of CT** |  |
| Diagnostic CT | CT performed at the emergency department, out-patient clinic or ward to diagnose the fracture |
| Calibration CT (3D scan) | 3D scan performed in the hybrid surgical theatre at the beginning of the operation to match the diagnostic CT – in which the screws are planned – to the intraoperative situation |
| Hybrid 3D CT (3D scan) | 3D scan performed during or at the end of the operation in the hybrid surgical theatre to check the position of the previously inserted screws, cement or K-wires. |
|  |  |
| **Types of screws** |  |
| SI Screw S1 with augmentation | 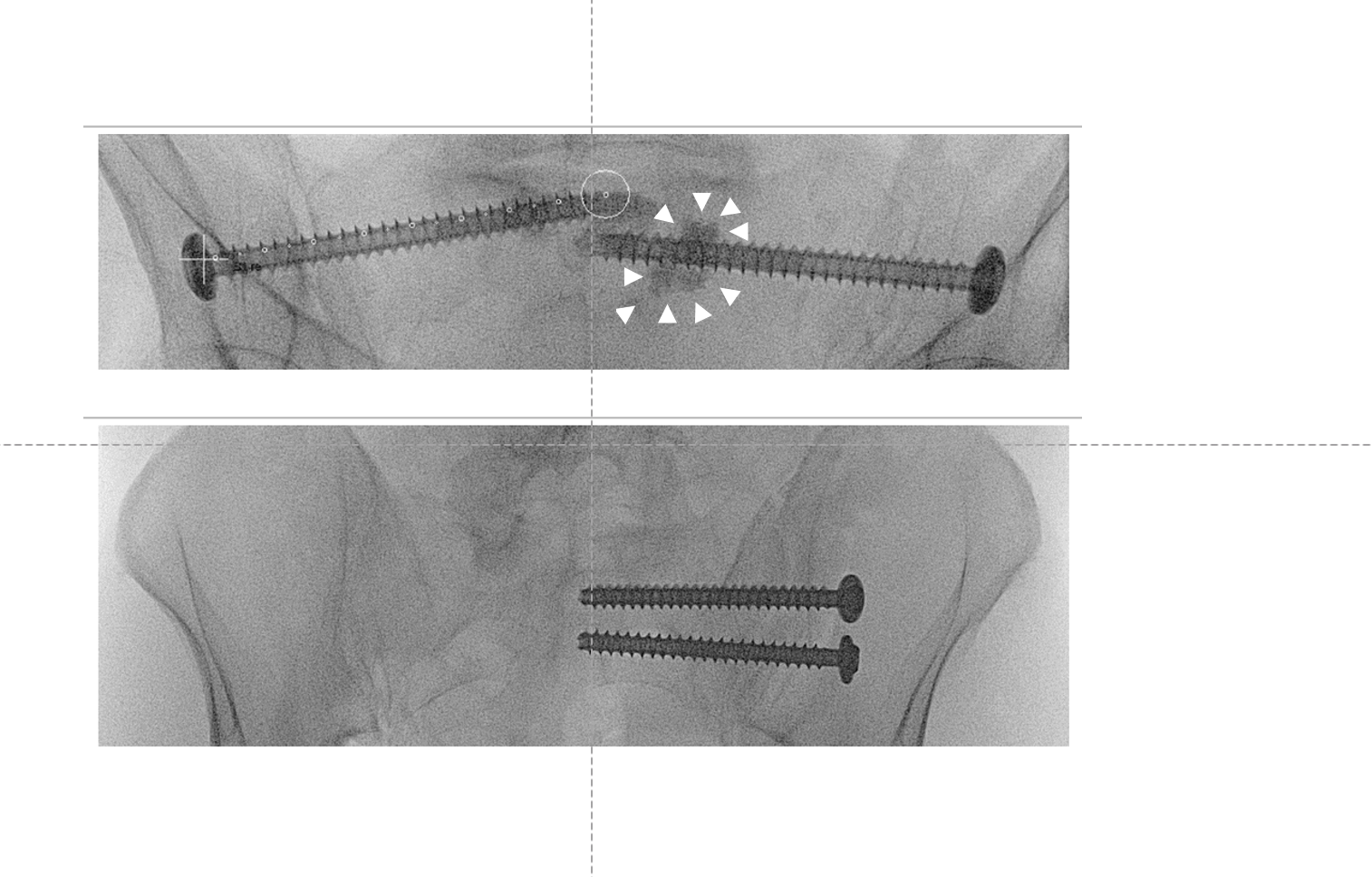 |
| SI Screw S2 without augmentation | 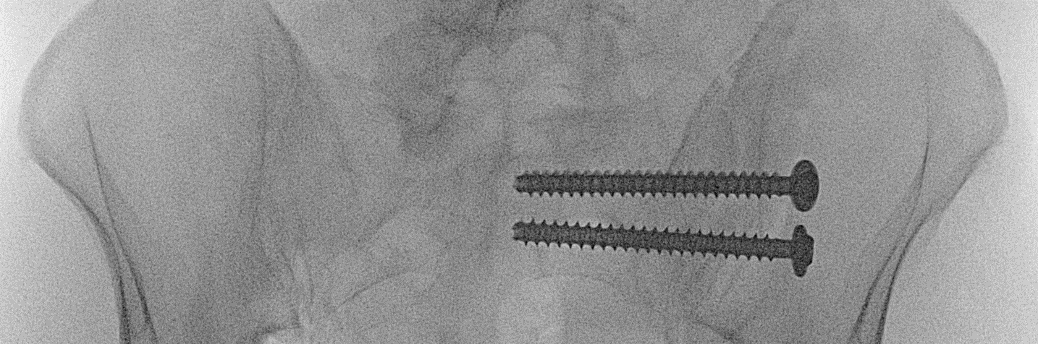 |
| Retrograde superior ramus screw | 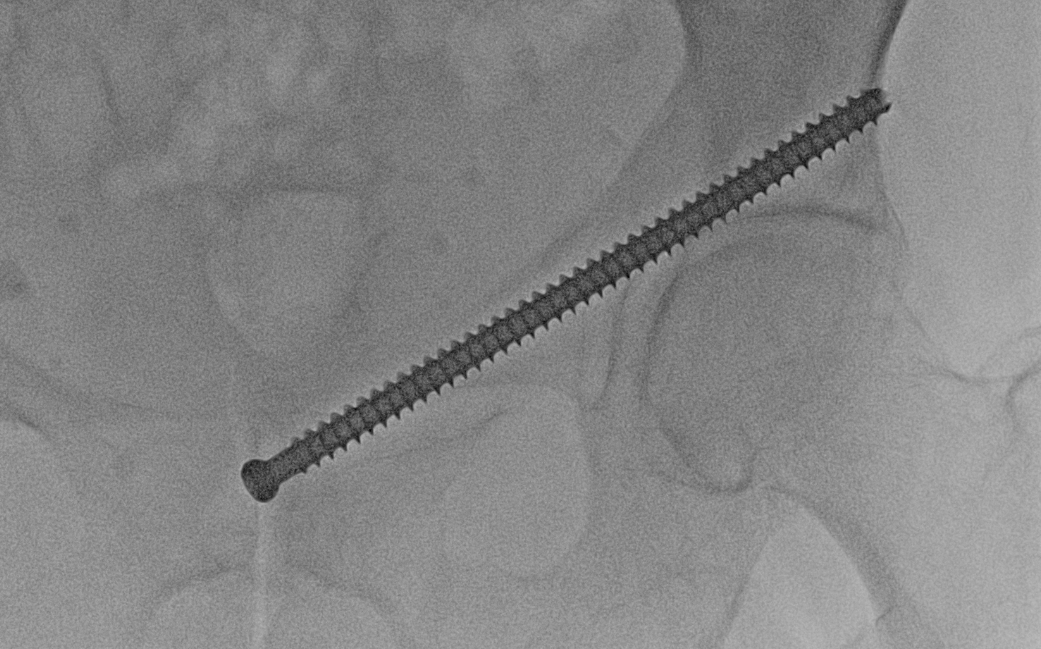 |
| Transiliosacral screw | 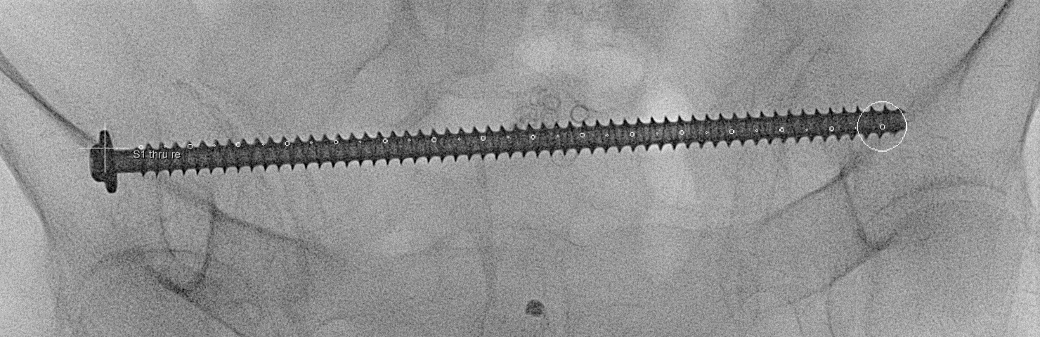 |
| Sacral bar | 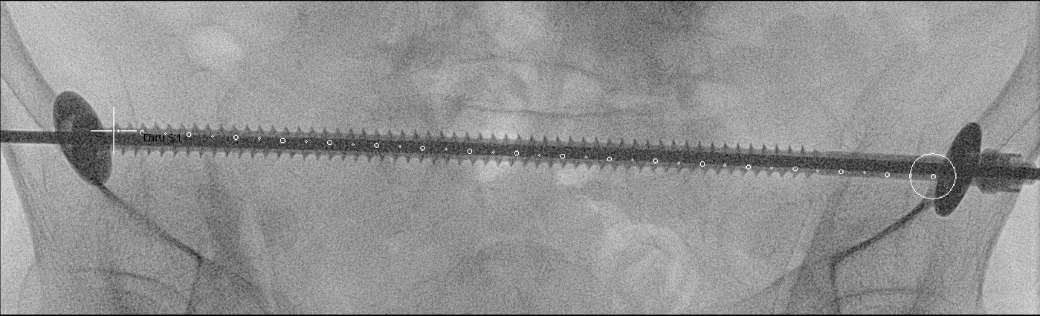 |
